# Intracranial administration of deglycosylated C-terminal-specific anti-Aβ antibody efficiently clears amyloid plaques without activating microglia in amyloid-depositing transgenic mice

**DOI:** 10.1186/1742-2094-3-11

**Published:** 2006-05-10

**Authors:** Niki C Carty, Donna M Wilcock, Arnon Rosenthal, Jan Grimm, Jaume Pons, Victoria Ronan, Paul E Gottschall, Marcia N Gordon, Dave Morgan

**Affiliations:** 1 Alzheimer's Research Laboratory, University of South Florida, Department of Molecular Pharmacology and Physiology, 12901 Bruce B Downs Blvd, Tampa, FL 33612, USA; 2 Rinat Neuroscience Corp. 3155 Porter Drive, Palo Alto, California, 94304, USA

## Abstract

**Background:**

Antibodies against the Aß peptide clear Aß deposits when injected intracranially. Deglycosylated antibodies have reduced effector functions compared to their intact counterparts, potentially avoiding immune activation.

**Methods:**

Deglycosylated or intact C-terminal specific high affinity anti-Aβ antibody (2H6) were intracranially injected into the right frontal cortex and hippocampus of amyloid precursor protein (APP) transgenic mice. The untreated left hemisphere was used to normalize for the extent of amyloid deposition present in each mouse. Control transgenic mice were injected with an antibody against a drosophila-specific protein (amnesiac). Tissues were examined for brain amyloid deposition and microglial responses 3 days after the injection.

**Results:**

The deglycosylated 2H6 antibody had lower affinity for several murine Fcγ receptors and human complement than intact 2H6 without a change in affinity for Aß. Immunohistochemistry for Aβ and thioflavine-S staining revealed that both diffuse and compact deposits were reduced by both antibodies. In animals treated with the intact 2H6 antibody, a significant increase in Fcγ-receptor II/III immunostaining was observed compared to animals treated with the control IgG antibody. No increase in Fcγ-receptor II/III was found with the deglycosylated 2H6 antibody. Immunostaining for the microglial activation marker CD45 demonstrated a similar trend.

**Conclusion:**

These findings suggest that the deglycosylated 2H6 is capable of removing both compact and diffuse plaques without activating microglia. Thus, antibodies with reduced effector functions may clear amyloid without concomitant immune activation when tested as immunotherapy for Alzheimer's disease.

## Introduction

The molecular mechanisms underlying Alzheimer's disease (AD) have been extensively investigated. AD can occur as a result of genetic mutations in the genes encoding presenilin 1, presenilin 2, or amyloid precursor protein (APP). These genetic alterations accelerate the pathological characteristics of AD, including the formation of extracellular amyloid plaques and the formation of intracellular neurofibrillary tangles consisting of hyperphosphorylated *tau*. The accumulation of these amyloid plaques are not only a crucial factor in the pathology of AD [1], but have been argued to contribute to the distinctive clinical symptoms of AD such as progressive cognitive decline, loss of memory and decreased mental capacity [2,3]. Consequently, reducing β-amyloid (Aβ) in brain has been a primary focus in the treatment of Alzheimer's disease.

Active immunizations using Aβ _1–42 _vaccine was first described by Schenk et al. (1999). This demonstrated that immunotherapy could be a successful means of significantly reducing Aβ deposits in amyloid depositing PDAPP transgenic mice. Not only have vaccinations with Aβ _1–42 _been shown to prevent plaque formation when initiated before the onset of amyloid deposit formation but can also reduce pre-existing brain amyloid [4]. Moreover, Janus et al. and Morgan et al. [5,6] demonstrated that vaccines against Aß could also protect APP transgenic mice from developing memory impairments. These observations initiated clinical trails in which patients with mild to moderate AD were given an active immunization (AN1792); [7-9]. These Phase IIa trials were interrupted due to the occurrence of meningoencephalitis in 6% of the patients [10].

Consequently, passive immunization became considered as a possibly safer and more controllable means of removing Aβ deposits from the brain. Immunization with anti-Aβ monoclonal antibodies has been demonstrated to be an efficient and effective means of clearing Aβ plaques with both prolonged systemic administration and intracranial injections of antibody [11-14]. In addition, passive immunization rapidly reversed cognitive deficits and memory loss in amyloid depositing transgenic mouse models [15,16].

Despite the initial promise of passive immunization as effective and practical treatment for AD, recent studies have demonstrated potentially harmful aspects of Aβ passive immunotherapy in mouse models of amyloid deposition. In several experiments administration of at least two different monoclonal anti-Aβ IgG's resulted in significant increases in occurrence and severity of cerebral hemorrhage when compared to controls [17-19]. Wilcock et al. [18] also showed an increase of cerebral amyloid angiopathy (CAA) in association with increases in vascular leakage. Microglial activation has been shown surrounding amyloid-containing blood vessels following systemic passive immunization and could potentially be one of the mechanisms that increase the likelihood of microhemorrhage [18].

In the present study we investigate the efficacy of a modified (deglycosylated) antibody with decreased affinity for the Fcγ receptor (Fcγ-R; [20]) for its ability to eliminate Aβ from the brain without increasing microglial activation. This will inform us if future passive immunization studies may use this modification to clear Aβ without activating microglia, and test the role of the microglial activation through Fcγ-R activation on vascular amyloid deposition and increased susceptibility to microhemorrhage.

## Materials and methods

### Antibody preparation

Antibody 2H6 is raised against aa33–40 of human Aß. The antibody binds Aß terminating at position 40 preferentially over peptides ending at position 42 and is of the murine IgG2b isotype. To generate deglycosylated 2H6 (de-2H6), N-linked carbohydrate groups on the Fc portion of the antibody were enzymatically removed by treatment with peptide-N-glycosidase F (QA-Bio, San Mateo). The antibody was incubated for 7-days at 37°C; with 0.05 U of enzyme per mg of antibody in 20 mM Tris-HCl pH 8.0; 0.01% Tween. The deglycosylated antibody was protein A purified and endotoxin was removed by Q-Sepharose anion exchange chromatography. Complete removal of N-linked glycans was verified by MALDI-TOF-MS and protein gel electrophoresis.

Binding affinity of 2H6 and de-2H6 antibodies to Fcγ receptors or complement protein C1q were also measured using BIAcore. Purified murine Fcγ receptors (from R&D Systems) and human C1q (from Quidel) were immobilized on BIAcore CM5 chip by amine chemistry: Fcγ receptors or C1q were diluted into 10 mM sodium acetate pH 4.0 and injected over an EDC/NHS activated chip at a concentration of 0.005 mg/mL. Variable flow time across the individual chip channels were used to obtain 2000–3000 response units (RU). The chip was blocked with ethanolamine. Serial dilutions of monoclonal antibodies (ranging from 2 nM to 70 μm) were injected. HBS-EP (0.01 M HEPES, pH 7.4, 0.15 M NaCl, 3 mM EDTA, 0.005% Surfactant P20) was used as running and sample buffer. Regeneration studies showed that a mixture of Pierce elution buffer (Product No. 21004, Pierce Biotechnology, Rockford, IL) and 4 M NaCl (2:1) effectively removed the bound antibody peptide while keeping the activity of Fcγ receptors and C1q. Binding affinities of Aß for the antibodies was determined similarly by immobilizing the antibodies on a CM5 chip using amine chemistry, and flowing AB1-40 over the chip at multiple concentrations. Binding data were analyzed using 1:1 Langmuir interaction model for high affinity interactions, or steady state affinity model for low affinity interactions.

### Experimental design

Transgenic mice. Tg2576 APP mice [2]) were acquired from the breeding colonies at the University of South Florida. Multiple mice were housed together whenever possible until the time of use for the study; mice were then singly housed just before surgical procedures until the time of sacrifice. Study animals were given water and food (*ad libitum*) and maintained on the twelve hour light/dark cycle and standard vivarium conditions. Two cohorts of mice were used, the first cohort consisted of mice aged 20 months (*n *= 13) and the second cohort consisted of mice aged 13 months (*n *= 15). Animals in each cohort were assigned to one of three groups. Group one received a C-terminal high affinity anti-Aβ antibody 2H6 (Rinat Neurosciences, Palo Alto, CA; *n *= 12; five 20 mo and seven 13 mo). Group two received de-2H6 antibody (Rinat Neuroscience; *n *= 8; four 20 mo and four 13 mo). Group three received a control antibody (also isotype IgG2b), directed against a drosophila protein, amnesiac, without a mammalian homologue (2908, Rinat Neuroscience) (*n *= 8; four 20 mo and four 13 mo). Overall measures of Aß load and Thioflavin S load were greater in the older mice.. Although there was a trend for greater fractional reductions of Aß by 2H6 and de-2H6 in younger mice, these observations were not consistent. Fractional reduction of Thioflavine S staining by antibodies was unaffected by the age of the mouse.

### Surgical procedure

Immediately before surgery mice were weighed then anesthetized using isoflurane. Surgery was performed on animals using a stereotaxic apparatus. The cranium was exposed using an incision through the skin along the median sagittal plane, and two holes were drilled through the cranium over the right frontal cortex injection site and the right hippocampal injection site. Previously determined coordinates for burr holes, taken from bregma were as follows; frontal cortex, anteroposterior, -1.5 mm; lateral, -2.0 mm, vertical, 3.0 mm, hippocampus, anteroposterior, -2.7 mm; lateral -2.5 mm, vertical, 3.0 mm. Burr holes were drilled using a dental drill bit (SSW HP-3, SSWhite Burs Inc., Lakewood, NJ). Injections of 2 μg antibody in 2 μl saline were dispensed into hippocampus and frontal cortex over a period of 4 min. using a 26 gauge needle attached to a 10 μl syringe (Hamilton Co., Reno, NV). The incision was then cleaned and closed with surgical staples. Animals were recovered within 10 minutes and housed singly until time of sacrifice.

### Immunohistochemistry

Three days post surgery, mice were weighed, overdosed with pentobarbital (200 mg/kg;) and perfused with 25 ml of 0.9% normal saline solution then 50 ml of freshly prepared 4% paraformaldehyde. Brains were collected from the animals immediately following perfusion and immersion fixed in 4% paraformaldehyde for 24 hrs. Mouse brains were cryoprotected in successive incubations in 10%, 20%, 30% solutions of sucrose; 24 hrs in each solution. Subsequently, brains were frozen on a cold stage and sectioned in the horizontal plane (25 μm thickness) on a sliding microtome and stored in Dulbecco's phosphate buffered saline (DPBS) with 0.2% sodium azide solution at 4°C.

Six sections 100 μm apart spanning the site of injection were chosen and free-floating immunochemical and histological analysis was performed to determine total Aβ using a rabbit anti-Aß serum at a concentration 1:10,000 (Serotec, Raleigh, NC), CD45 expression using rat anti-mouse monoclonal IgG; 1:5000 (Serotec, Raleigh, NC), and Fcγ-receptor-II/III (Fcγ-R) expression using rat anti-mouse monoclonal IgG; 1:1000 (BD Biosciences, San Diego, CA). A fourth series of sections were mounted on slides and stained with thioflavine-S (1%; Sigma Aldrich, St. Louis, MO) to assess compact plaque deposition. Immunohistochemical procedural methods were analogous to those described by Gordon et al. 2002 for each marker. Six sections from each animal were placed in multisample staining tray and endogenous peroxidase blocked (10% methanol, 30% H_2_0_2, _in PBS). Tissue samples were then permeabilized (with lysine 0.2%, 1% Triton X-100 in PBS solution), and incubated overnight in appropriate primary antibody. Sections were washed in PBS then incubated in corresponding biotinylated secondary antibody (Vector Laboratories, Burlingame, CA). The tissue was again washed after a 2 hr. incubation period and incubated with Vectastin^® ^Elite^® ^ABC kit (Vector Laboratories, Burlingame, CA) for enzyme conjugation. Finally, sections were stained using 0.05% diaminobenzidine and 0.3% H_2_0_2 _(for CD45 and FcγR 0.5% nickelous ammonium sulfate was added for color enhancement). Tissue sections were mounted onto slides, dehydrated, and coverslipped. Each immunochemical assay omitted some sections from primary antibody incubation period to evaluate nonspecific reaction of the secondary antibody.

Stained sections were imaged using an Evolution MP digital camera mounted on an Olympus BX51 microscope at 100 × final magnification (10 × objective). Six horizontal brain sections (100 μm apart; every 4^th ^section) were taken from each animal and four nonoverlapping images near the site of injection from each of these sections were captured (24 measurements per mouse). All images were taken from the same location in all animals. Quantification of positive staining product surrounding and including the injection sites in the right frontal cortex and the right hippocampus and the corresponding regions in the left hemisphere were determined using Image-Pro^® ^Plus (Media Cybernetics^®^, Silver Springs, MD). Ratios of the right and left regions were calculated (to normalize for variability in amyloid deposition between animals) and ANOVA statistical analysis was performed using StatView^® ^version 5.0.1 (SAS Institute, Raleigh, NC).

## Results

### Antibody deglycosylation

**Figure 1:**
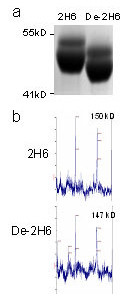
Verification of deglycosylation of de-2H6 by and MALDI-TOF-MS and SDS-PAGE. Panel A. SDS-PAGE analysis of 2H6 and de-2H6. Samples were size fractionated under denaturing conditions on a 3–8% Tris-Acetate Gel and stained with Coomassie blue. Note the lower apparent molecular weight for the deglycosylated heavy chain doublet. Panel B. MALDI-TOF-MS analysis revealed the expected 2% reduction in molecular weight after removal of N-linked glycans in the de-2H6 antibody

The treatment with peptide-N-glycosidase F appeared to completely remove the single carbohydrate chain associated with the Fc component of IgG for antibody 2H6. This was apparent both by mobility shift on polyacrylamide gel analysis of the denatured IgG heavy chain (Fig 1a) and by a shift in molecular weight by MALDI-TOF analysis of the native IgG complex (Fig 1b).

Deglycosylation had no effect on the affinity of 2H6 for its antigen, Aß1–40, but exhibited reduced binding to its effector proteins responsible at least in part for the activation of microglia and other cells in association with antigen opsonization (table 1).

**Table 1:** Affinities (Kd) of 2H6 and De-2H6 for antigen and effector proteins

Antibody	Aß1–40 (nM)	mFcγRI (μM)	mFcγRIIb (μM)	mFcγRIII (μM)	hC1q (μM)
2H6	8	1.6	20	39	5
De-2H6	9	6.5	30	67	30

### Amyloid clearance

**Figure 2:**
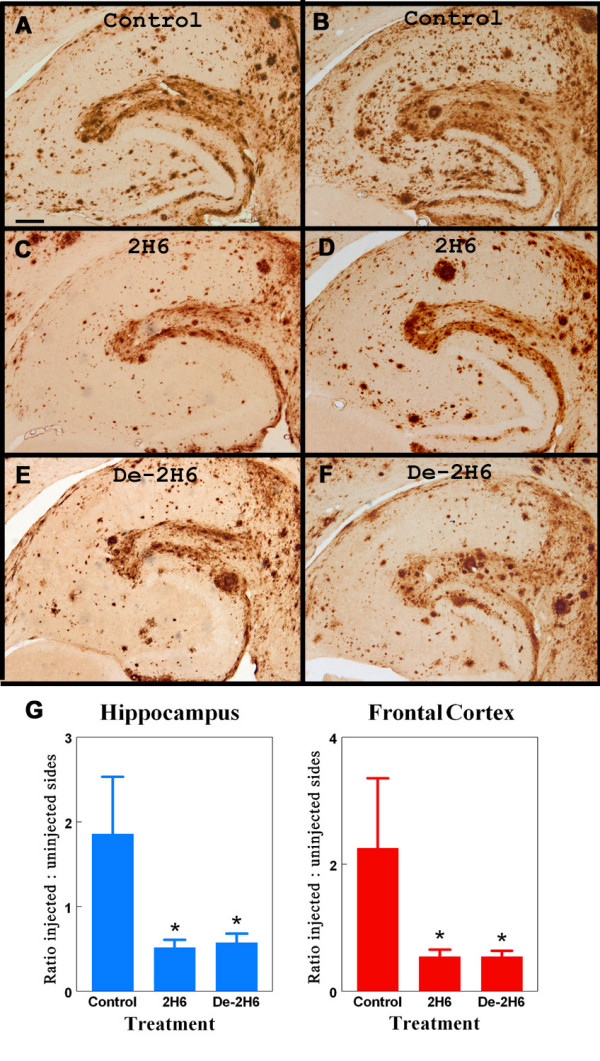
Total Aß load is reduced following intracranial administration of intact anti-Aβ antibody and deglycosylated anti-Aβ antibody. Panels B, D, and F show total Aβ immunostaining in the left (untreated) hippocampal regions of 20 mo. old APP transgenic mice. Panels A, C, and E show total Aβ staining in right hippocampal regions of 20 mo. old APP transgenic mice receiving intracranial injection of control antibody (panel A) or anti-Aβ C-terminal antibody (2H6; panel C), or deglycosylated anti-Aβ C-terminal antibody (de-2H6; panel E). Magnification = 40×, scale bar = 50 mm. Panel G shows quantification of the Aß load as the ratio of injected (right) side to uninjected (left) side for both the hippocampal and frontal cortical injection sites * indicates P < 0.05 compared to mice injected with control IgG.

Intracranial injections of the intact 2H6 antibody, de-2H6 antibody and control IgG were administered to APP mice and immunohistochemistry was performed on fixed brain tissue to determine amount of plaque clearance. Total Aβ load was ascertained 3 days after intracranial injections by immunohistochemical methods using a polyclonal anti Aβ antiserum which primarily recognizes the N-terminal domain of Aß, and thus labels both Aβ _1–40 _and Aβ _1–42 _(the time course of Aβ clearance and diffusion patterns of injected anti-Aβ antibodies were presented by Wilcock et al., (2003)[14]). The regional Aβ distribution and density in APP transgenic mice were similar to those reported by Gordon et al. and Hsiao et al. [21,2]. Immunohistochemistry revealed darkly stained compact plaques and more lightly stained diffuse plaque deposits containing fibrillar and nonfibrillar β-amyloid in the APP animal tissue (Fig 2A). Plaque deposition was distributed throughout the cortical regions as well as in the hippocampus (although most concentrated in the molecular layers of the dentate gyrus and the CA1 region, surrounding the hippocampal fissure). A notable decrease in the amount of hippocampal Aβ staining was observed in animals injected with intact 2H6 and de-2H6 antibodies (Fig. 2C and 2E) 72 hrs after time of injection in comparison to control animals receiving the anti-amnesiac IgG (Fig 2A). Animals injected with the control antibody showed Aβ immunohistochemical staining patterns throughout the cortex and hippocampus comparable to those of untreated APP transgenic mice of the same age. The reductions in Aβ deposition were limited to the areas surrounding the cortical and hippocampal injection sites. ANOVA analysis of animals injected with the intact 2H6 IgG showed significant reduction (72%) in the hippocampus and a significant reduction (76%) in the frontal cortex compared to animals treated with the control IgG (Fig. 2G). Mice treated with the de-2H6 showed significant reductions in both the hippocampus (69%) and in the frontal cortex (76%). In neither region was there a difference between mice treated with 2H6 compared to mice treated with de-2H6.

**Figure 3:**
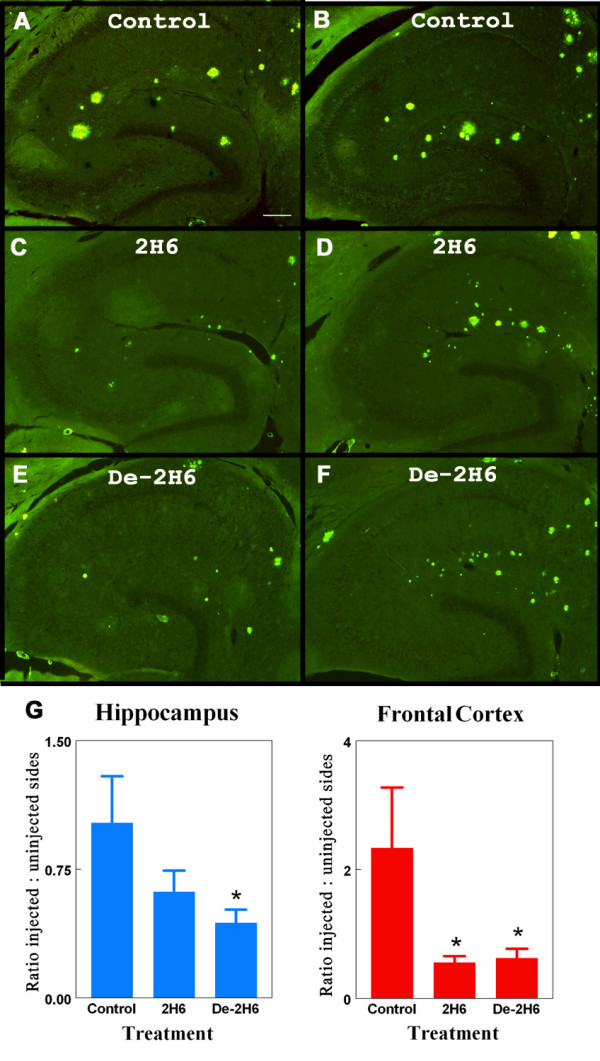
Thioflavine S labeled compact amyloid deposits are reduced following intracranial administration of anti-Aβ antibody. Panels B, D, and F show total thioflavine S staining of compact amyloid deposits in left (untreated) hippocampal regions of 20 mo. old APP transgenic mice. Panels A, C, and E show total thioflavine S staining in right hippocampal regions of 20 mo. old APP transgenic mice receiving intracranial injection of control antibody (panel A) or anti-Aβ C-terminal antibody (2H6; panel C), or deglycosylated anti-Aβ C-terminal antibody (de-2H6; panel E). Magnification = 40×, scale bar = 50 μm. Panel G shows quantification of the amyloid load as the ratio of injected (right) side to uninjected (left) side for both the hippocampal and frontal cortical injection sites * indicates P < 0.05 compared to mice injected with control IgG.

As noted by our previous work [14] thioflavine-S staining labels compact fibrillar amyloid plaques, but not the more diffuse Aβ staining. The thioflavine-S positive plaque deposition was homogeneously distributed throughout the frontal cortical regions, but in hippocampus was concentrated along the hippocampal fissure and into the dentate gyrus (Fig 3A). The density of thioflavine-S staining was substantially less than Aβ immunochemistry staining. Antibody administration reduced thioflavine-S positive staining three days after antibody administration (Fig. 3C and 3E). Quantification of positive staining at the site of injection in animals receiving the de-2H6 anti-Aβ antibody showed a significant reduction (55%) in the hippocampus and a significant reduction (70%) in the frontal cortex compared to mice injected with the control antibody (Fig 3G). Injection of intact 2H6 caused significant reduction (75%) in positive staining in the frontal cortex, but the 35% reduction in hippocampal plaque load did not reach significance compared to the control antibody values (Fig. 2G). Again, no differences were found when the intact and the deglycosylated anti-Aß antibody groups were compared. Vascular Aβ levels were calculated by measuring thioflavine S stained area after digitally editing out parenchymal (plaque) deposits. No significant changes in vascular Aβ were seen with the intact or deglycosylated anti-Aβ antibody groups when compared to control animals.

### Microglial activation

**Figure 4:**
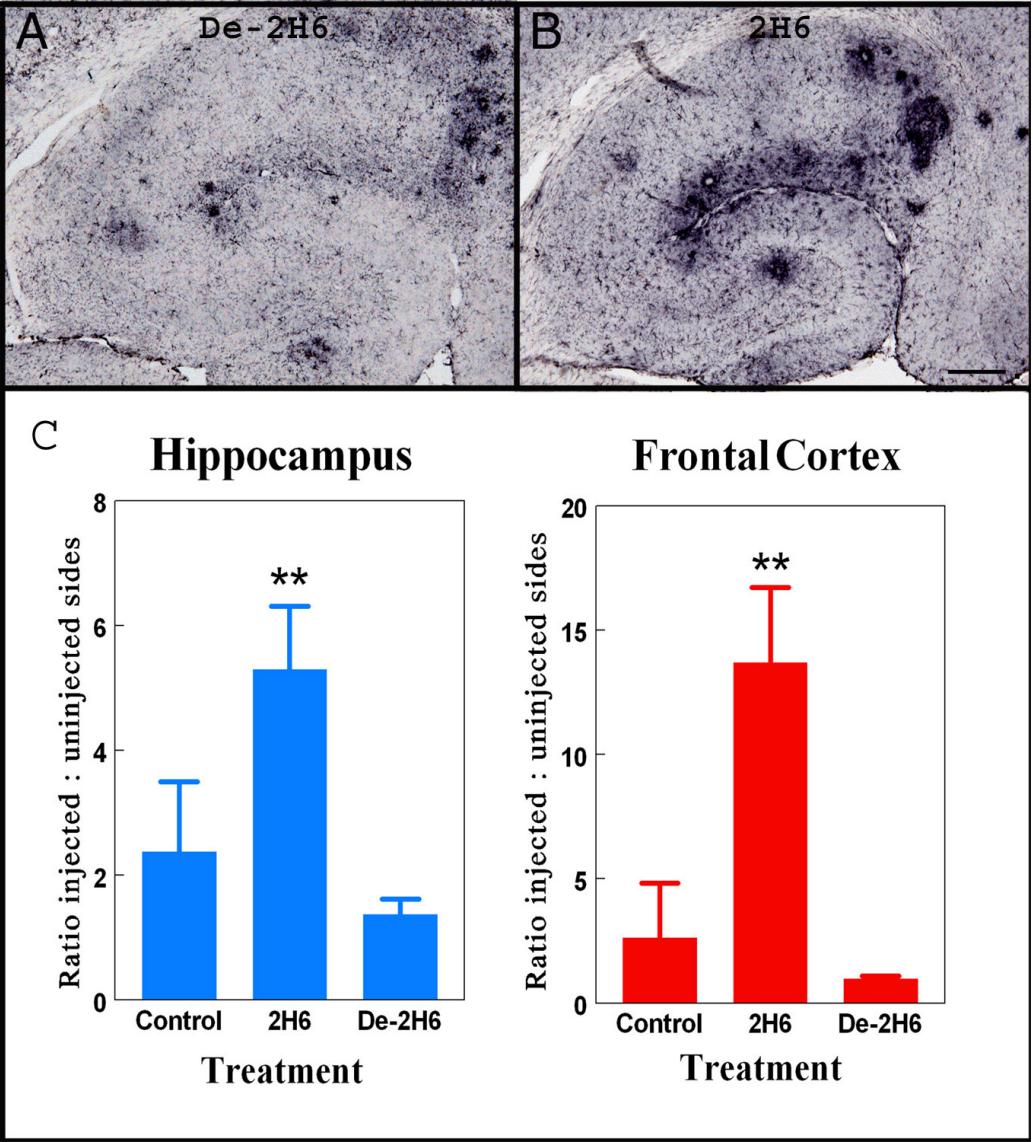
Fcγ receptor expression is increased following intracranial administration of intact anti-Aβ antibody but not deglycosylated anti-Aβ antibody. Panels A and B show Fcγ-receptor II/III staining in right hippocampal regions of 20 mo. old APP transgenic mice receiving intracranial injection of deglycosylated C-terminal anti-Aβ antibody (de-2H6) (panel A) or intact anti-Aβ C-terminal antibody (2H6; panel B). Magnification = 40×, scale bar = 50 μm. Panel C shows quantification of the Fcγ-R immunostaining as the ratio of injected (right) side to uninjected (left) side for both the hippocampal and frontal cortical injection sites. ** Indicates P < 0.01 versus both control IgG and de-2H6.

After determining efficacy of 2H6 and de-2H6 were similar in clearance of both diffuse and compact Aβ, we examined microglial activation by looking at Fcγ-R expression and CD45 expression. In prior work we found that antibody opsonized antigens in brain increase microglial expression of Fcγ-R, presumably to aid in phagocytosis of the opsonized material [22]. The staining patterns in animals injected with de-2H6 and control antibody were similar to that of untreated APP transgenic mice (Fig. 4A). All animals demonstrated the most intense activation in areas immediately surrounding Aβ plaques within the dentate gyrus and near the fissure. Fcγ-R immunohistochemistry for mice receiving the intact 2H6 antibody was increased considerably both near the amyloid deposits, and to a lesser extent throughout the hippocampus (Fig 4B). Quantification and ANOVA analysis of Fcγ-R expression levels revealed a significant fivefold increase in the frontal cortex and hippocampus in animals receiving the 2H6 antibody compared to mice receiving either the control anti-amnesiac IgG or the de-2H6. In contrast, the group receiving intracranial administration of de-2H6 showed no changes in Fcγ-R expression when compared to the control antibody group (Fig. 4C).

**Figure 5:**
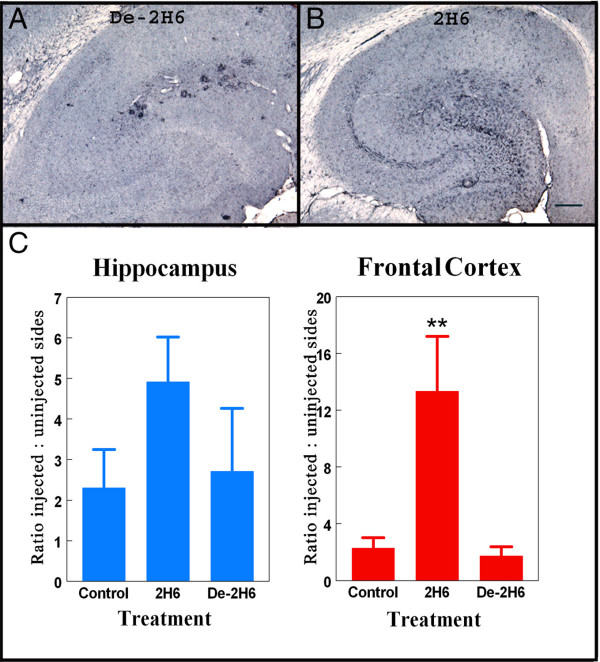
CD45 expression is increased in mice receiving intracranial administration of intact but not deglycosylated anti-Aβ antibody. Panels A and B show total CD45 staining in right hippocampal regions of 20 mo. old APP transgenic mice receiving intracranial injection of deglycosylated C-terminal anti-Aβ antibody (de-2H6) (panel A) or intact anti-Aβ C-terminal antibody (2H6; panel B). Magnification = 40×, scale bar = 50 μm. Panel C shows quantification of theCD45 immunostaining as the ratio of injected (right) side to uninjected (left) side for both the hippocampal and frontal cortical injection sites * indicates P < 0.05 compared to mice injected with control IgG and de-2H6.

The staining patterns of the CD45 antibody were similar to patterns seen in tissue stained for Fcγ-R expression (Fig 5A). However, there was a slightly greater degree of microglial activity in the right hemisphere at the location of needle entry relative to the uninjected side due to mechanical injury from the injection procedure. In mice treated with the 2H6 antibody there was a significant elevation in activated microglia as detected by CD45 immunohistochemistry (Fig 5B.). Activated microglial patterns in animals treated with intact 2H6 were fairly widespread but slightly more concentrated staining was observed in areas immediately surrounding Aβ plaques as well as areas surrounding the sites of injection in the frontal cortex and hippocampus (Fig. 5B). Quantitative analysis showed a dramatic increase, approximately sixfold, in microglial expression in the animals receiving 2H6 compared to those animals receiving control antibody or the de-2H6 in frontal cortex (Fig 5C). A less dramatic, but similar trend was observed in the hippocampus. Brains injected with the de-2H6 showed no significant changes compared to control mice, and were significantly lower than the mice injected with intact 2H6 in the frontal cortex 3 days after treatment (Fig 5A; 5C).

## Discussion

The formation and deposition of amyloid plaques composed largely of aggregated Aβ peptides is an invariant feature of AD, and several studies find inverse correlations with cognitive function [23-25]. There is a strong correlation between Aβ loads and cognitive function in APP transgenic mice [26,19,29]. A number of studies have demonstrated that passive immunization with anti-Aβ antibodies can remove considerable amounts of Aβ plaques in the amyloid depositing APP transgenic mice [11,30,14,31]. Immunotherapy with anti-Aß antibodies can also improve memory performance in amyloid depositing APP mice [6,5,15,16,18]. The data from this study demonstrate that intracranial administration of either an anti-Aβ antibody which exhibits high affinity for the C terminal of the Aβ peptide or its deglycosylated counterpart (with an impaired ability to bind to the Fc effector proteins) provide effective methods by which to remove Aβ. Both anti-Aβ antibodies illustrated a considerable capacity to reduce both compact and diffuse Aβ plaque pathology. There were no significant differences between the capacities of these antibodies to remove Aß deposits in spite of the reduced effector activating functions of the deglycosylated variant. It is possible that near the site of injection, most of the removable Aß deposits are cleared by both antibodies. This could suggest that some limit in the extent of clearance is reached rather than suggesting activated microglia have no role in antibody-mediated Aß clearance.

The effectiveness of passive Aβ immunotherapy in reversing AD brain pathology raises questions concerning the underlying mechanisms by which anti-Aβ antibodies produce such dramatic reductions in Aβ in the brain. Some results argue that amyloid opsonization and Fcγ-R mediated phagocytosis by microglia is the major mechanism by which Aβ is removed from the brain [4,11,14,32]. Other experiments, using the same intracranial approach described here, suggested that the activation of microglia can facilitate the removal of Aβ plaques in the brain, but may not be essential [22].

The observations presented in this study are more consistent with experiments indicating that microglia independent mechanisms can result in the efficient clearance of Aβ plaques. One such mechanism may involve the disruption of plaque by the antibody itself followed by disaggregation or disruption of the ß-sheet conformation of Aβ and subsequent removal [33]. Data presented by Bacskai et al. showed that F(ab')_2 _fragments (modified anti-Aβ antibody which lack the complete Fc region) were able to significantly decrease amyloid deposits following administration [12]. Additionally, Das et al. found that vaccination against Aß was able to significantly reduce amyloid deposition in Fcγ-R knock out mice lacking expression of Fcγ-RIII and possessing reduced phagocytic function [31]. Another mechanism referred to as the "peripheral sink" first described by DeMattos et al. [30] suggests that decreases in β-amyloid deposition following immunization is a result of the net efflux of Aβ from the brain to the plasma, facilitated by the antibody acting as a sink in the circulation, which then prevents further deposition of amyloid in the brain. A similar conclusion was drawn from work with another Aß binding agent, GM1 ganglioside, that increased plasma Aß and reduced central Aß deposition [34]. It is unlikely this latter alternative is at work in the studies with intracranial administration, but the catalytic disaggregation mechanism is certainly feasible with the intracranial approach. It is further conceivable that centrally applied antibodies may form a sink in the ventricular space of the brain, reducing parenchymal deposits [35]. Most recently, FcRn has been demonstrated to play a substantial role in amyloid removal by anti-Aß immunotherapy, by transporting both antibody into the brain, and antibody-Aß complexes out of the brain[18]. Thus, there are several mechanisms by which anti-Aß immunotherapy may function without requiring activation of effector proteins and activation of microglia or other immune cells. Recent studies indicate that deglycosylation does not affect the capacity for the antibody to bind to the neonatal Fc transport receptor (FcRn;[36]).

The efficacy and success of anti-Aβ immunotherapy in the treatment of amyloid pathology (reducing Aβ plaque load and reversing or halting cognitive decline) in both mice and humans [7,8,37,38], despite some drawbacks [10], has initiated further exploration into the cellular responses underlying removal of Aß in the brain. Recent experiments with prolonged systemic passive immunization have revealed adverse affects including increases in microhemorrhage in transgenic mice accompanied by reductions in diffuse and fibrillar amyloid [39,17,19]. A link between increases in vascular amyloid levels and increases in cerebral hemorrhage following passive immunization was also reported recently [39]. The precise mechanism by which passive immunotherapy leads to increased levels of hemorrhage has not been clearly delineated but it has been proposed that antibody opsonization of vascular amyloid may activate local microglia to produce an inflammatory response [17,19]. Additionally, the increases in vascular amyloid levels following passive immunization may result from microglial mediated redistribution of compacted amyloid from the parenchyma to the vessels, further weakening the blood vessels leading to increased susceptibility to cerebral hemorrhage [39]. Regardless of the cause of increased risk of hemorrhage, minimizing the interaction between passively transferred anti-Aß antibodies and effector proteins on the microglial surface may have benefits with respect to microhemorrhage development.

The deglyosylated anti-Aβ antibody used in this study is a modified version of the high affinity C-terminal Aß40-specific 2H6 antibody in which the carbohydrate groups within the Fc portion of the antibody have been removed, significantly impairing its ability to bind to the Fcγ-receptors of macrophages and, presumably, reducing Fc mediated phagocytosis. A similar effect of deglycosylation on an N terminal specific anti-Aß antibody was recently reported in vitro [40]. Even though recent trials have exposed some adverse consequences of one Aβ vaccine, the benefits of immunotherapy as a potential treatment for Alzheimer's disease should not be undervalued. The present results suggest that the modified deglycosylated antibody provides an efficient means of removing Aβ from the brain without activating microglia. Emphasis on further exploration into the mechanisms involved in antibody mediated Aβ removal from the brain and elucidation of more effective methods of immunotherapy continues to be an important area of focus in AD therapy.

## Competing interests

A. Rosenthal, J. Grimm and J. Pons are employees and shareholders of Rinat Neurosciences Corporation which holds the patents for the antibodies used in the studies presented here. D. Wilcock has also performed consulting services for Rinat Neurosciences.

## Authors' contributions

Niki Carty performed the surgical procedures, histological measurements and data analysis. She also drafted the first version of the manuscript. Donna Wilcock supervised the surgical procedures and assisted in the histology. Arnon Rosenthal, Jaume Pons and Jan Grimm developed the 2H6 monoclonal antibody and produced the material for injection. Jaume Pons performed the deglycosylation procedure and measured affinities using the Biacore. Victoria Ronan was responsible for all genotyping of transgenic mice and assisted in maintenance the mouse colony. Paul Gottschall prepared the polyclonal antiserum used for histological measurement of Aß and assisted in manuscript preparation. Marcia Gordon was responsible for tissue collection and data analysis. Dave Morgan was responsible for overseeing all aspects of the study and played the major role in manuscript revision.
